# The True Physician

**Published:** 1892-11

**Authors:** 


### The True Physician.

Dr. T. Frazer Thomas, of Gainesville, Florida, is the author of the following sentiment touching the relations of the medical man to the lowlier members of his constituency: "The true physician will respect the feelings of the poor, both by the language and tone of voice in which he addresses them. He will remember that disease is his only passport to any house. He will act as a gentlemen to all, to the low, to the vile even, as well as the gentle and the rich. His duty is to heal, not to punish." Boerhaave said that "the poor were the best patients, for God is their paymaster." Because the physician receives no tangible recompense he must not forget his obligation to his patient nor his own self-respect.

In his intercourse with the world he must not be swayed by prejudice nor nationality. Friendship and good-will for all his patients are his polar stars, ever keeping in remembrance the priceless precept, "There is but one country—the earth; but one nation—the human race."

The principles set forth in the above lines, contain the gist, in a certain direction, of the true professional character.

It applies to the specialist as well as to the general practitioner, and with a special force does it apply to the dentist.—Ed.

